# Editorial: Proton magnetic resonance spectroscopy in brain aging: Inflammation, bloodflow, connectivity and cognitive decline

**DOI:** 10.3389/fpsyt.2022.1040967

**Published:** 2022-10-10

**Authors:** Antoine Hone-Blanchet, William Vallet, Salman Shahid, Gabrielle Ende

**Affiliations:** ^1^Department of Radiology, Athinoula A. Martinos Center for Biomedical Imaging, Harvard Medical School, Massachusetts General Hospital, Boston, MA, United States; ^2^INSERM U1028, CNRS UMR 5292, PSYR2 Team, Centre de recherche en Neurosciences de Lyon (CRNL), Universite Lyon 1, Lyon, France; ^3^School of Medicine, Indiana University Bloomington, Bloomington, IN, United States; ^4^Central Institute of Mental Health, Medical Faculty Mannheim, University of Heidelberg, Mannheim, Germany

**Keywords:** magnetic resonance imaging, spectroscopy, brain aging, GABA, inflammation

In the past 20 years, proton magnetic resonance spectroscopy (MRS) has developed into an established and robust imaging method to gather critical neurochemical information *in vivo* in the human central nervous system. Estimation of metabolites concentrations has proven beneficial in several clinical conditions, including age-related neurodegenerative disorders, and normal physiological brain aging. In healthy and pathological brain aging, significant physiological changes include cortical atrophy, impairment of bloodflow and glucose metabolism, and elevated inflammation; which eventually lead to more severe structural but also functional and behavioral changes. All of these root causes for physiological brain aging have a direct impact on neurochemistry; concentration levels of metabolites thus become key proxy markers for such physiological modulations. Taken together, this positions modern MRS as an extremely valuable imaging modality to assess neurological state, but also the therapeutic impact of a large range of interventions. In this Research Topic, we sought to collect novel contributions to the field from MRS researchers working either directly on the use of MRS and additional imaging modalities in the study of brain aging, or in development of MRS imaging in the perspective of furthering research in brain aging.

A crucial issue in brain aging, and one that has arguably stoked much interest in MRS research, is inflammation and, incidentally, the term coined inflammaging. Normal age-related inflammation is considered one of the main factors for development of neurodegenerative disorders. In their paper, Vints et al. assessed the interaction between MRS metabolites obtained from several regions of interest and blood-derived peripheral inflammatory markers interleukin-6 and kynurenine. They show that elevated levels of kynurenine were associated with increased levels of choline and myo-inositol in the left somatosensory and dorsolateral prefrontal cortices, suggesting a relationship between peripheral and cortical neuroinflammation.

In another publication from the Research Topic, Krishnamurthy L. C. et al. found that by combining MRS with pseudo-continuous arterial spin labeling MRI, coupling of Glx (glutamate + glutamine) and cerebral blood flow was significantly lower in healthy older adults compared to young adults. This suggests a potential role for altered glial activation in older adults, and differential roles for Glx, GABA, and CBF in the study of the aging brain in the context of upper extremity motor performance. Further, the positive coupling between cardiorespiratory fitness (as quantified by VO_2_max) and metabolite concentrations GABA and Glx suggests that higher fitness allows for a more efficient metabolic shift that facilitates improved performance on cognitive-motor tasks.

In another study combining MRS with task-based blood oxygen level-dependent (BOLD) MRI, Krishnamurthy V. et al. investigated the age differences in performance of a language task. They found that, although resting levels of GABA and Glx did not predict task-BOLD amplitude, GABA related to the start of the task block that was less difficult while Glx related to the end of the task block that was more difficult. Importantly, they report that GABA and Glx may have a push-pull relationship with vascular factors (i.e., dispersion of the BOLD hemodynamic response function) derived from task-fMRI, indicating that inhibitory and excitatory metabolites may influence neurovascular coupling differentially. This elicits the importance of combining neurovascular-natured BOLD and MRS in the study of aging, and shows that multi-modal assessments of the neurophysiology may help tease apart the neural and vascular contributions of the BOLD signal in an aging-related context.

Besides the study of glutamate and GABA, metabolites like glutathione, the main antioxidant of the human central nervous system, are also of crucial interest in the study of physiological brain aging. Accordingly, novel approaches to reliably estimate levels of glutathione have been at the forefront of MRS technical developments in recent years. In their paper, Song et al. compared peak-fitting approaches to glutathione quantification with HERMES and MEGA-PRESS acquisition sequences. They show that linear combination modeling improved reliability of glutathione quantification for HERMES data, providing additional guidelines for future utilization of this very sequence.

Finally, although this Research Topic has mostly focused on brain aging, it is important to mention the important body of work on neurodegenerative disorders which has been using MRS as main imaging modality. Importantly, some of those works have focused on the identification early biomarkers for Alzheimer's disease [e.g., (1, 2)], but also in several other conditions, including Parkinson's disease. In their meta-analysis, Gu et al. have investigated data in several studies on MRS markers for early diagnosis of Parkinson's disease in subcortical areas substantia nigra and globus pallidus. They report that the ratio of n-acetylaspartate to creatine showed a significant difference between patients with early-stage Parkinson's disease and healthy controls. This suggests obvious neurochemical modulation in subcortical nuclei with depletion of dopaminergic neurons, but also that MRS could provide additional metrics in the evaluation and identification of early-stage Parkinson's disease.

Taken together, the works presented in this Research Topic demonstrate recent advancements in MRS, and other imaging modalities, in the study of brain aging and related pathologies. Such advances advocate for (1) the relevance of MRS in the field and (2) the importance of the continuing effort in developing MRS in the study of the aging brain.

## Author contributions

All authors listed have made a substantial, direct, and intellectual contribution to the work and approved it for publication.

## Conflict of interest

The authors declare that the research was conducted in the absence of any commercial or financial relationships that could be construed as a potential conflict of interest.

## Publisher's note

All claims expressed in this article are solely those of the authors and do not necessarily represent those of their affiliated organizations, or those of the publisher, the editors and the reviewers. Any product that may be evaluated in this article, or claim that may be made by its manufacturer, is not guaranteed or endorsed by the publisher.
